# Attitudes towards sub-domains of professionalism in medical education: defining social accountability in the globalizing world

**Published:** 2017-04-20

**Authors:** David Ponka, Douglas Archibald, Jessica Ngan, Brendan Wong, Sharon Johnston

**Affiliations:** 1 Department of Family Medicine, University of Ottawa, Ontario, Canada

## Abstract

**Background:**

Unmet health needs of populations around the world are a major contributor to lagging health outcomes globally. Medical professionals have a duty to address the health needs of their communities. In a globalizing world, the needs may seem limitless. Yet, most training involves immersion in one health system and its resources. How do practitioners reconcile this potentially limitless demand with their focused training and in understanding their duty to the populations they serve?

**Methods:**

A mixed-method design was used. We distributing a pre-validated survey examining all facets of professionalism to the Department of Family Medicine at the University of Ottawa. This was followed by interviewing a purposive sample of residents and faculty with different levels of interest in working with underserved populations, to examine attitudes towards social accountability.

**Results:**

Quantitative results did not replicate the factor structure of the pre-validated survey in our cohort. This and other gaps in individual responses were used to construct an interview guide. Interviews revealed differences between residents and faculty. Residents were likely to see social accountability as flowing from personal interest as opposed to a professional duty; and residents’ sense of duty can be met through good care of individual patients under their sphere of care. Faculty were more likely to discuss facets of care that they could influence at the health system level nationally and beyond.

**Conclusion:**

More usable and succinct instruments are needed to capture individual attitudes on social accountability. Our results identify how new physicians in family medicine in Ottawa, Canada wish to apply learning in global health to local needs, responding to the call to “think global, act local.”

## Introduction

Over the past years, global health needs have been highlighted by the Ebola epidemic in Africa, the Zika virus outbreak affecting South America, and the Syrian civil war causing the largest number of displaced people since World War II. How the medical community responds to these catastrophes in our globalizing world, and how it defines the limits of its involvement is very much of interest to those involved in studying the evolution of medical professionalism.

Professionalism is a guiding code for physicians established both by the profession and the communities it serves, and whose content accordingly evolves with changing social expectations and needs. The Royal College of Physicians and Surgeons of Canada CanMEDS framework^1^ states that “as Professionals, physicians are committed to the health and well-being of individual patients and society through ethical practice, high personal standards of behaviour, accountability to the profession and society, physician-led regulation, and maintenance of personal health.” There is little controversy about such a definition. What is more variable is an individual physician’s interpretation, understanding, and embodiment of these principles.

For the past 20 years or so, academics have tried to tie the evolution of professionalism alongside that of evolving human health needs. Charles Boelen among others^2^–^5^ and the World Health Organisation (WHO) have been leading a call to make medical schools more accountable to society. Cruess and Cruess have reminded us that professionalism forms the very basis of the social contract that physicians have with society.^6^ More recently, the CanMEDS/Royal College Expert Working Group on Professionalism has refined its definition by adding responsiveness to societal needs and expectations.^1^ The Association of Medical Colleges of Canada (AFMC)^7^ goes even further, reminding us of our responsibility to the “global community.”

Assessing professionalism at the trainee level is therefore gaining more attention as the importance of professionalism for medicine’s mission is both recognized and developed. Studies have established that the professional behaviours of learners are multidimensional and require multiple tools to assess.^8^,^9^ Cohen^10^ adds to the complexity by noting the important difference between professional behaviours and the attitudes that underpin them. He writes, “[p]rofessionalism denotes a way of behaving in accordance with certain normative values, whereas humanism denotes an intrinsic set of deep-seated convictions about one’s obligations toward others.”

In other words, how one *understands* the various aspects of professionalism, as opposed to how one merely demonstrates professional *behaviours*, regardless of underlying attitudes (without which behaviours are much less likely to be sustained), is an area of study that is gaining interest, and is the focus of this paper.

At the time of planning the study we conducted a literature review on objectifying attitudes towards medical professionalism and could find few pre-validated instruments for this purpose. Tsai et al.^11^ conducted a factor analysis of “latent factors” (underlying attitudes) behind professionalism amongst trainees in Taiwan, but this may not be generalizable to North American providers, for several reasons. First, residents and faculty were not included in this study. More important, as the authors state, Taiwan is only now emerging from a culture where volume of patients seen and patient outcomes are considered primordial values of the profession. Morreale^12^ compared resident and faculty attitudes towards social responsibility, but did so by using a non-validated survey. Ho^13^ attempted to do the same, but that study also occurred in Taiwan, and used reactions to video clips that again are difficult to compare across cultures.

Blackall et al.^14^ performed another factor analysis study at Penn State University Medical School among residents and faculty, our population of interest. They asked participants to rate the six *a priori* American Board of Internal Medicine (ABIM)^15^ components of professionalism for importance and relatedness. Their analysis led to seven independent dimensions with acceptable internal consistency reliability estimates (Cronbach’s alpha values given in parentheses): accountability (0.77), altruism (0.73), duty (0.71), enrichment (0.78), equity (0.71), honor and integrity (0.71), and respect (0.51).

The aim of our study was to better understand if the sub-domains of professionalism, such as the ones outlined by ABIM and in Blackall’s instrument are equally well-understood and emphasized within one academic department of family medicine, in Ottawa, Canada. We further sought to identify among a single diverse cohort how family physicians understand the concept of social accountability and how they perceive it is most effectively learned and taught. Finally, we wanted to elucidate any differences in this understanding between residents and faculty.

## Methods

Sequential, exploratory mixed-methods approaches^16^ are comprised of two-phases: a quantitative phase and a qualitative phase. The results of the quantitative portion of the study permit more refined questions to be asked during subsequent interviews, and for clarifications to be sought. We therefore first distributed on online survey to all members of the Department of Family Medicine (DFM) at the University of Ottawa (both faculty and residents) and, based on the results, constructed an interview guide for the qualitative portion. This approach allowed us more in-depth exploration with the interviewees.

### Context

At the time of the study the DFM was a large department composed of 210 residents and 395 faculty members. Of these faculty members, 45 were core faculty, working at mostly urban sites, some of which are more inner-city and have more of a tradition of outreach to underserved communities, including the care of the homeless and refugee populations. Among residents, approximately 60% were female and 17% were International Medical Graduates (IMGs). DFM faculty were recently offered a professionalism course, entitled “Essential Teaching Skills 3,” which is focused on remediation of unprofessional behaviours. The DFM also gave residents the option of enrolling in a global health stream, to work with underserved communities both locally and overseas. Traditionally there were clinical teaching units within DFM that embraced this stream more openly than others. We therefore used a purposive method of sampling to interview respondents from all these different facets of the program.

### Quantitative phase: survey

Blackall’s Penn State Professionalism Questionnaire (PSPQ) (permission given by author to use the instrument) was transformed into an online version in Fluid Surveys**™**. An email invitation was extended to residents (n=210) and faculty (n=395) within the DFM at the University of Ottawa to gauge the level of importance they assigned the previously validated Penn State professionalism constructs. Individuals were asked to rate to what extent 36 statements describing various aspects of professional behaviours reflected their own definitions of professionalism, ranked from 1 (never) to 5 (great deal). The questionnaire included additional questions about perceived institutional social accountability, using Boelen & Heck’s^3^ conceptual grid, in the areas of educational, research and clinical activities, in order to give us a baseline understanding of how members of the DFM reflected on the importance of their institution responding to the broader needs of society. A draw for a VISA gift card was used as incentive for survey participation and multiple reminders were sent over a period of eight weeks.

Confirmatory factor analysis was performed in R (package lavaan) by an experienced statistician to check the factor structure offered in Blackall et al.’s original paper. An average “Professionalism Attitude Score” (PAS) was then calculated from the ratings provided for questions under each construct. Further, averages were identified by demographic descriptors (gender, year of entry, International Medical Graduate/Canadian Medical Graduate, resident/faculty, having taken a recently offered professionalism course (ETS3) for faculty, and global health stream enrollment for residents).

### Qualitative phase: interviews

For the qualitative phase we constructed an interview guide using data generated from the first phase of this project and major trends as described in the literature review. We then invited key informants from the DFM. Respondents were purposively chosen to capture many different viewpoints within the DFM from all four major family medicine teaching sites in Ottawa, both junior and senior residents, and junior and senior faculty across both inner-city and more suburban sites (since inner-city sites tend to have a higher proportion of homeless and other disadvantaged patients). Among residents, we interviewed residents who were enrolled in our Global Health stream as well as residents who were not. Finally, we selected both Canadian Medical Graduates (CMGs) and IMGs from both residents and faculty pools. With support from an assistant, two authors (JN and DA) interviewed six residents and six faculty. Analysis commenced between interviews and we reached theme saturation after these 12 interviews. Individual interviews were conducted either in person or over the phone, an alternative that has been shown acceptable in most situations.^17^ Interviews were recorded and transcribed. Each participant received a $20 coffee shop gift card.

The interviews were professionally transcribed and thematically analyzed individually by three authors (JN, DP, SJ) using the immersion/crystallization technique.^18^ An *a priori* coding framework was generated using concepts and data from the quantitative phase and from the literature review and this framework was revised as new important themes emerged, based on team discussion. The authors agreed on the final coding template, transcripts were coded by one of the authors (JN) and indexed individually, and a thematic chart generated for the whole data set, from which representative quotes were selected.

Ethics approval was given by the Ottawa Health Sciences Network Research Ethics Board.

## Results

### Quantitative phase (Survey)

The survey respondents were representative of the overall composition of the DFM (Table 1). Thirty-five resident respondents and 13 faculty respondents were obtained, with an additional 15 respondents who did not specify, for a total of 63 respondents. The average response time for fully completed questionnaires was 18.75 minutes.

We attempted to replicate Blackall et al.’s theoretical factor structure based on our responses but were unable to do so. The model failed to converge, that is, the responses did not group themselves under the predicted professionalism constructs.

**Figure 1:**
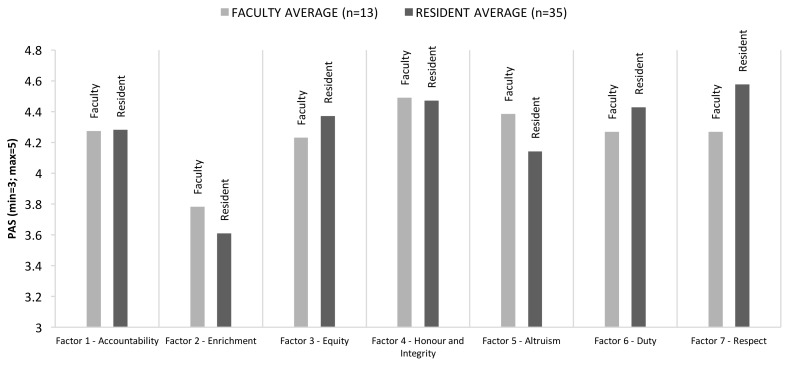
Attitudes towards professionalism factors across various family medicine cohorts. Mean scores are depicted. PAS=Professionalism Attitude Score, scored on a scale of 0–5.

Figure 1 illustrates our Professionalism Attitudes Score (PAS) across various cohorts within the DFM. Several interesting findings emerged. The professionalism factor of “enrichment” in Blackall’s study (a newly validated factor based on those responses that “showed a willingness to initiate and offer assistance toward a colleague’s professional and personal development”) was rated less important by our respondents, across cohorts.

No significant differences between residents and faculty were detected in the responses but there were several other interesting findings. Faculty who took a recently offered faculty development course at the DFM—the Essential Teaching Skills 3: Professionalism—ranked all the factors with a higher PAS than faculty who did not take the course (data for this subgroup and the next not shown). Among residents, those who were IMGs ranked factors similarly to CMGs, except for Factor 5: altruism. IMGs ranked this factor much lower than their CMG counterparts.

Table 2 illustrates the perceived institutional (departmental) delivery of the three social accountability mandates according to various family medicine cohorts.

Although all three mandates were rated highly, respondents differed in their assessment of the relative merit of the DFMs contribution: faculty were more likely to rate the educational and research mandates highly and residents were more likely to rate the clinical mandate highly. This was especially true for male resident in our cohort. Finally, faculty who took the ETS 3: Professionalism course at the DFM were much more likely to rate all mandates highly.

### Qualitative phase (Interviews)

The survey was not able to elicit the specific relationships between professionalism constructs and what the limits of our engagement should be. It was also not able to detect significant differences in attitudes between residents and faculty. We thus constructed an interview guide which focused on these areas for the qualitative portion of our study. We then conducted semi-structured interviews with six residents and six faculty from diverse backgrounds and from different units, as described in the methods. Three recurrent themes emerged.

#### The relationship between professionalism, social accountability, and global health

All interview participants appreciated the interrelation between the concepts of professionalism, social accountability and global health as the interviews progressed, although residents in particular initially struggled to define the concepts and the distinctions between them.

*Social accountability? Um, being accountable for I don’t know, let me think. Is it more than maintaining patient confidentiality? I think so.* (Resident, Canadian Medical Graduate (CMG), non-Global Health (GH) stream)

There tended to be variability around the definition of global health, especially between faculty and residents. In response to the question “have you had any prior interest or experience in global health or in practicing in under-serviced areas?”, one faculty respondent answered:

*Under-serviced yes, global no.* (Faculty, had taken Essentials Teaching Skills 3 (ETS3), non-GH site)

Whereas a resident responded:

*I think we should always strive to be socially accountable and whether or not it is someone in front of us or someone in a different country I don’t think that there is really a difference. I think that they are related to each other one is just I think more like outside of my country. I feel like family medicine is global health, especially where we are practicing right now. We are frequently dealing with people of different cultures, nations, languages and so I feel like social accountability should exist for both. I feel like I am practicing global health actually, where I am.* (Resident, CMG, non-GH)

When asked to define the concept of professionalism, participants focused on the individual patient encounter. Residents in particular tended to limit their responses to concepts of responsibility and respectfulness in the clinical setting, without considering the interface with wider community needs:

*[Professionalism is] being on time and being respectful of my patient and my colleagues, being reliable and responsible for the tasks given to me.* (Resident, CMG, non-GH)

The understanding of social accountability and its relationship to professionalism did evolve after definitions were given:

*I think the more socially accountable you are, the more professional you are.* (Resident, CMG, non-GH).

However, a focus on individual patient care remained even towards the conclusion of interviews:

*I don’t know I think we set the example for our patients and by being socially accountable I think it helps to form those long lasting patient relationships, I think it helps to form trust, respect from patient to doctor but also like doctor to doctor their colleagues and other health professionals. I think that that is why it is important.* (Resident, CMG, non-GH)

The discussion turned next to how best to develop social accountability, a specific facet of professionalism, within the DFM.

#### Enablers and detractors to developing social accountability

Participants identified that the concept of social accountability wasn’t necessarily formally taught in the curriculum, making it difficult for residents to recognize it as an important competency in professionalism.

*I feel like they don’t really teach social accountability…we will talk a bit more on specific populations. But even I think relative to other things in the curriculum, probably not that much…I mean, the fact that we are talking about this and I don’t know what the definition is kind of speaks to that.* (Resident, CMG, non-GH)

*We sort of took for granted that people would know [what professionalism was] without realizing that people may have to learn the essence of it.* (Faculty, ETS3, GH site)

Many examples of how social accountability can be taught were provided, including dedicated classroom time and small group teaching. It was felt that the most effective method was through faculty role modeling. One resident was able to reflect on clinical experiences with a preceptor who exhibited social accountability in her practice.

*I definitely see some of our preceptors being socially accountable and I think maybe seeing them has taught me more about being socially accountable than maybe what I have been formally taught[…] But my preceptor when there was that earthquake in Nepal, she specially trained in obstetrics and she went to Nepal to help with maternity care there. I feel that that is an example of social accountability on a global scale. She does that here as well in one of the underserved maternity clinics or the clinics for young moms, which I think is another example of social accountability. So she doesn’t necessarily teach it but she shows it.”* (Resident, CMG, non-GH)

Likewise, a faculty member, identified the importance of modeling professional behaviours to residents.

*We would model professionalism in how we interact with our resident on a daily basis, what emphasis we put on staying late enough to do what has to be done, and not sort of rushing off before the is done, or coming in on time ourselves and showing a patient first attitude.* (Faculty, ETS3, GH site)

Respondents engaged keenly on the limits of our collective professional responsibility. A key limit in terms of scope was the sheer volume of work to be done. Both residents and faculty most frequently expressed potential burnout as a barrier, or the fear of being spread too thin or of being overworked to the point that it affected other areas of practice.

*I don’t want [working with underservice populations] to take up my whole practice, I find it too draining. I feel like I can’t be a good physician.* (Resident, CMG, non-GH)

*If you are so used up that you can’t be fully there for people then you are not doing them a service. So part of being professional is knowing what your limits are, when the red flag comes up saying you have done too much and it is time for a break.* (Faculty, ETS3, GH site)

Residents consistently expressed the idea that social accountability needs to come from personal interest, as opposed to a sense of duty:

*Do I think you have to have an interest in it? Yes. I think you need to…to have to desire to be socially accountable…* (Resident, CMG, non-GH)

The implications of the thinking behind social accountability arising from individual interests and not from current professional frameworks, are important.

#### Scope of social accountability

As respondents did not naturally reflect on other limits to the scope of engagement, beyond time barriers and personal interest, we also asked questions about their understanding of “societal need.”

Table 3 summarizes the focus of residents’ and faculty’s discourse around this line of questioning. As respondents reflected on the concepts presented in the interviews, residents tended to interpret social accountability more at a local level.

Although faculty also referred to local needs, they were much more likely to spend time discussing overseas initiatives, including new partnerships meant to increase the internationalization (or partnerships with the aim of exchanging information, global influence and the sharing of research agendas, as opposed to the care of underserved populations) of our institution.

Notably, residents were eager to discuss applying lessons across the local-global divide:

*If we were to enhance our global health initiative, we need to make sure to always tie it back and say “This is what is happening there, but it is also actually still happening here* [in Canada].” *And so there* [are] *similarities and we need to try to solve some of the problems here and at the same time be altruistic and try to get to some of these other communities that need help that are outside of Canada as well.* (Resident, International Medical Graduate (IMG), non-GH)

One resident in particular was keen to discuss to issue of underserved areas of Quebec, directly across the border from Ottawa

*And in Ottawa specifically there seems to be a definite lack of resources on the Quebec side and a lot of resentment towards people coming from Quebec for Ottawa resources. One example I saw of that was in labour and delivering unit where one woman was almost turned away because she was from Quebec. I don’t know being from Alberta where we are not on a border we don’t deal with this kind of stuff so it just seemed like the most ridiculous thing. So maybe one issue in Ottawa that I see as an outsider is that accessibility to health care is threatened, which is supposable something that is foundational to Canadian health care.* (Resident, CMG, GH)

Several other residents mentioned the relative under-serviced nature of Western Quebec as a social accountability problem, but no faculty did.

## Discussion

We were not able to replicate Blackall et al.’s^14^ findings of seven independent professionalism constructs. The exploratory analyses we performed in our cohort suggest a different factor structure. We would have expected to see greater concordance with Blackall, despite the smaller sample, if indeed the instrument is well suited to distinguishing these features.

While a larger sample size may have led to a more significant result, we would have expected some replicability to come from a pre-validated instrument such as the PSPQ, even with a small sample. Such instruments, after all, may have to be used on more limited samples or within smaller institutions. It is possible that such instruments need more validation studies across jurisdictions, when cultural and other differences may be at play. Recently, Davis and Reyes^15^ published a synthesis package on the PSPQ suggesting reliability across settings but calling into question another factor that may explain our results: the possibility that a survey that is reliable in paper form may not be so online. Further study of an online version of the PSPQ is required.

The findings of instrument validity not necessarily extending from paper to digital formats, as well as our low survey response rate, lead us to call for the development and validation of more streamlined and online instruments to probe for attitudes around, and constructs of, professionalism among medical learners and faculty. It was not unusual for our respondents to need over 20 minutes to complete the 36 item PSPQ. Interestingly, even though our respondents rated “enrichment” (of their colleagues’ interests and careers) lower than other factors, they nonetheless took the time to complete our lengthy survey. It may be however, that the under-emphasis on “enrichment” in part explains the poor response rate to research surveys generally in the medical community.^20^

In the survey phase of our study members of the DFM rated the importance of most professionalism factors highly and their perceived institutional social accountability highly prompting more detailed questioning in our interviews. Interestingly, social accountability scores may simply reflect a cohort’s interests—and specifically their emphasis on the importance of professionalism. For example, faculty who did not take a recently offered course on professionalism were less likely to rank the importance of all professionalism factors highly, and were less likely to rank institutional efforts highly. This underscores Boelen and Heck’s^3^ call to “initiate and stimulate reform in medical schools,” through initiatives meant to highlight professionalism and social accountability. This call was made 20 years ago, but is a work in progress.

From the interviews, our most interesting finding was that of a generational difference between residents and faculty with regards to the scope of social accountability. Specifically, residents seemed to wish to engage in international initiatives to better understand problems, but then apply this knowledge at a local level. Though our faculty did not discount local needs, they were more likely to be interested in sustained engagement internationally or in response to international problems.

This finding is consistent with the literature on generational differences in professionalism. Most of this literature notes that trainees, when reflecting on professionalism, focus on their unmet learning needs—i.e. mastering immediate patient care rather than more distant system or community issues.^21^

Other studies describe generational differences in how social accountability is understood. Morreale et al.^12^ found in a pilot study that residents in a psychiatric residency program ranked social responsibility significantly more highly than did faculty. Ho et al.^13^ found that altruism and accountability were more important constructs to residents compared to faculty in Taiwan, and that residents’ perceptions more closely matched those of standardized patients and patient advocates.

However, Boelen and Woollard^4^ differentiate social accountability, which is an ultimate goal, from social responsibility, wherein priorities are community-oriented but defined by the institution, and social responsiveness, which is an intermediate step where priorities are data-driven and outcome-based. Social accountability goes a step further by aspiring to a shared agenda-setting with communities, and by aiming for sustained impact. Future research should probe respondents around these subtly different concepts.

Our additional finding that, among trainees, the impetus for social accountability may come from individual interest rather than an overarching professional framework has significant implications for training programs and specifically admission committees. This finding merits further confirmation and study.

A limitation of this study is that there is currently no agreed upon or specific measure of an individual’s construct of social accountability. As Tsai^11^ has pointed out, there is a lot of variability in the understanding of professionalism constructs such as ABIM’s. This understanding may also be cohort dependent, explaining in part the lack of replicability of Penn State findings in a Canadian cohort. Currently, social accountability can be variably understood as a composite of ABIM’s “duty,” “accountability” and “equity” constructs, but this needs further study to validate questions aimed at gauging an individual’s commitment to the needs of their communities or the wider society. We hope to have added to this important body of work.

Another potential limitation for the project is that for the interview phase of the project we may not have fully sampled key informants within the DFM. For example, we were not successful in securing even one interview from the francophone stream of our program. This cohort would be important to explore further, especially in light of the International Conference of Francophone Deans and Faculties of Medicine’s (ICFDFM) recent consensus statement, which highlights social accountability as a “priority strategic orientation.”^22^

### Conclusion

Our study was a cross-sectional analysis of attitudes surrounding social accountability as a unique aspect of professionalism, in a Canadian Department of Family Medicine, and suggests possible intergenerational differences. The incoming generation of medical practitioners is more likely to be influenced by personal interest when choosing how to respond to the needs of society. On a more optimistic note, they are also keen to apply lessons learned with an international lens onto local needs, truly heeding to the call to “think global, act local.”

Future research should consider a longitudinal study of similar nature, to understand whether these differences are truly intergenerational, or rather represent an evolution as medical practitioners move through their careers.

## Figures and Tables

**Table 1:** Demographic distribution of survey respondents

Cohort	n
Faculty	13
Residents	35
Female Residents	28
Male Residents	7
Residents 2013 Entry (PGY-2)	6
Residents 2014 Entry (PGY-1)	11
Residents 2015 Entry (Incoming PGY-1)	17
Full-time faculty	7
Community-based faculty	6
Faculty Who Have Taken “Essential Teaching Skills 3 – Professionalism” Course	7
Faculty Who Have Not Taken “Essential Teaching Skills 3 – Professionalism” Course	7
Residents Who Are in the Global Health Stream and/or Are Completing an Elective In Underserviced Community	18
Residents Who Are Not in the Global Health Stream Nor Are Completing an Elective In Underserviced Community	12
International Medical Graduates	6
Graduates from a Canadian Medical School	22
Total	63

**Table 2:** Perceived institutional delivery of social accountability mandates across family medicine cohorts. Scores out of a hundred on a sliding scale.

	Overall Average	**Faculty Average**	**Resident Average**	Female Resident Average	Male Resident Average	Faculty Who Have Taken “Essential Teaching Skills 3 – Professionalism” Course	Faculty Who Have Not Taken “Essential Teaching Skills 3 – Professionalism” Course
Education	67.18	**71.31**	**67.63**	70.32	56.86	78.71	62.67
Research	62.98	**70.54**	**61.85**	63.81	54.29	81.29	58.00
Clinical	72.55	**65.92**	**76.85**	75.15	83.43	76.14	54.14

**Table 3:** Representative quotes from residents and faculty around the ideal scope of social accountability

*Representative quotes*	Resident	Faculty
Focus:	*“I think that the program is super strong with work abroad, I haven’t necessarily seen a ton of work locally with marginalized populations…”*	“*Global health also has an internationalization component*.”
Example:	More local discourse: *“And in Ottawa specifically there seems to be a definite lack of resources on the Quebec side and a lot of resentment towards people coming from Quebec for Ottawa resources.”*	More global discourse: *“…the most obvious crisis at work especially right now with the Syrian refugees, 8 million individuals who are in kind displaced within their own county 4.5 million that are forced from their homes and they are now refugees living outside of their county of birth.”*
Applying lessons across the local-global divide :	*“Global health might be focusing on the health needs of under serviced areas so areas in Africa areas in Central and South America, some areas in Asia and sort of the deficiencies that they have in terms of supply and demand there. And it might not necessarily talk as much about what our social accountability in Canada is, or even our social accountability in the world, but I think probably once you are starting the conversation there it probably ends up getting tied back into where we are practicing here.”*	*“I mean one aspect of professionalism is looking at underserviced communities locally and globally but I think that you have to have a core understanding of professionalism for any facet of family medicine you are going to be in.”*
